# Pyogenic Sacroiliitis in Older Adults Presenting With Massive Retroperitoneal Abscess: A Case Report

**DOI:** 10.1155/cro/5538167

**Published:** 2026-10-17

**Authors:** Takahito Arai, Yasuchika Aoki, Masashi Sato, Yuki Akiyama, Kenta Inagaki, Takuya Sakamoto, Atsushi Hojo, Takayuki Fujiyoshi, Masahiro Inoue, Sumihisa Orita, Seiji Ohtori

**Affiliations:** ^1^ Department of Orthopedic Surgery Eastern Chiba Medical Center Togane Japan chiba-u.ac.jp; ^2^ Department of General Medical Science Graduate School of Medicine Chiba University Chiba Japan chiba-u.ac.jp; ^3^ Department of Orthopedic Surgery Kimitsu Chuo Hospital Kisarazu Japan hospital.kisarazu.chiba.jp; ^4^ Department of Orthopedic Surgery Graduate School of Medicine Chiba University Chiba Japan chiba-u.ac.jp; ^5^ Department of Medical Engineering Graduate School of Science and Engineering Chiba University Chiba Japan chiba-u.ac.jp

**Keywords:** case report, pyogenic sacroiliitis, retroperitoneal abscess, surgical drainage

## Abstract

Pyogenic sacroiliitis (PSI) is an uncommon form of septic arthritis and is frequently diagnosed late in older adults because initial symptoms are nonspecific and imaging findings may be misattributed to other sources of infection. We report two older patients with PSI complicated by massive retroperitoneal and gluteal abscesses, highlighting the importance of early recognition and, when indicated, timely surgical source control. Case 1 was an 83‐year‐old woman in whom a conspicuous retroperitoneal abscess and abnormal urinalysis led to suspicion of an intra‐abdominal or urinary tract source, delaying recognition of PSI. Despite antibiotics, she deteriorated with organ dysfunction; re‐evaluation of the initial magnetic resonance imaging MRI identified the sacroiliac joint as the primary focus, and staged surgical drainage procedures were required, followed by a prolonged recovery. Case 2 was a 70‐year‐old woman who presented in septic shock after failing outpatient treatment for presumed lumbar spine disease. Based on the diagnostic pitfalls in Case 1, PSI was immediately suspected; emergency drainage of both retroperitoneal and gluteal collections was performed on the day of presentation, which was followed by rapid stabilization and recovery. These cases emphasize that, in older patients with low back or gluteal pain, the concurrent presence of retroperitoneal and gluteal abscesses should prompt careful evaluation of the sacroiliac joint. These observations suggest that early diagnosis and timely assessment for surgical source control may contribute to favorable outcomes in severe cases.

## 1. Introduction

Pyogenic sacroiliitis (PSI) is an uncommon form of septic arthritis, accounting for approximately 1%–2% of all septic arthritis cases [1, 2], and can be difficult to diagnose promptly because early symptoms are often nonspecific [1]. These symptoms commonly include unilateral low back, lumbogluteal, or buttock pain and difficulty walking or bearing weight; fever and leukocytosis are variable, although inflammatory markers are usually elevated [2–5]. In older adults, low back or buttock pain is common and frequently attributed to benign musculoskeletal conditions, and systemic inflammatory signs may be variable, contributing to delayed recognition [2–5]. Reported predisposing contexts include injection drug use, pregnancy or the postpartum period, trauma, diabetes or other immunocompromising conditions, and preceding cutaneous, respiratory, or genitourinary infection, but many patients have no identifiable risk factor [2–5]. In particular, when a large abscess is the most conspicuous finding, clinical attention may be diverted away from the sacroiliac (SI) joint, and timely surgical intervention for source control may be postponed. Here, we report two older patients with PSI complicated by massive abscess formation: one case in which surgical intervention was delayed and another case in which earlier recognition and source‐control intervention, informed by the first case, were followed by a favorable clinical course.

## 2. Case Presentation

### 2.1. Case 1

An 83‐year‐old woman with a history of diabetes, hyperlipidemia, hypertension, thyroid tumor, spinal canal stenosis, and asthma presented with a 1‐week history of right‐sided low back pain. On presentation, her vital signs were body temperature 37.5°C, heart rate 110 bpm, respiratory rate 30 breaths/min, blood pressure 141/64 mmHg, and SpO2 91% on room air. Physical examination revealed erythema and swelling in the right gluteal region. Although it was difficult to exert force during hip joint movements due to pain, there was no other muscle weakness in the lower extremities, and reflexes were normal. Laboratory findings showed marked inflammation and hyperglycemia: total protein (TP) 5.3 g/dL, albumin (Alb) 1.9 g/dL, AST 23 U/L, ALT 39 U/L, CK 108 U/L, BUN 27.6 mg/dL, creatinine (Cre) 0.55 mg/dL, Na 131 mEq/L, K 4.1 mEq/L, Cl 100 mEq/L, CRP 31.07 mg/dL, glucose 497 mg/dL, HbA1c 6.9%, eGFR 78 mL/min/1.73 m2, WBC 16,520/*μ*L, RBC 354 × 10^4^/*μ*L, Hb 11.4 g/dL, platelets 22.0 × 10^4^/*μ*L, PT‐INR 0.99, APTT 26.8 s, and D‐dimer 2.3 *μ*g/mL. Urinalysis showed RBC 10–19/HPF, WBC > 100/HPF, and bacteria 3+. Magnetic resonance imaging (MRI) demonstrated widening of the affected SI joint space with fluid collection, along with edema signal changes in the surrounding muscles, sacrum, and ilium, as well as extensive abscesses in the retroperitoneal space lateral to the iliopsoas, gluteal region, and paraspinal muscles (Figure 1). The Day 0 MRI was formally interpreted by a radiologist after the day of presentation, and the report raised the possibility of PSI. However, this interpretation was not incorporated into the immediate treatment plan; PSI was recognized when the initial MRI was subsequently re‐evaluated.

**Figure 1:**
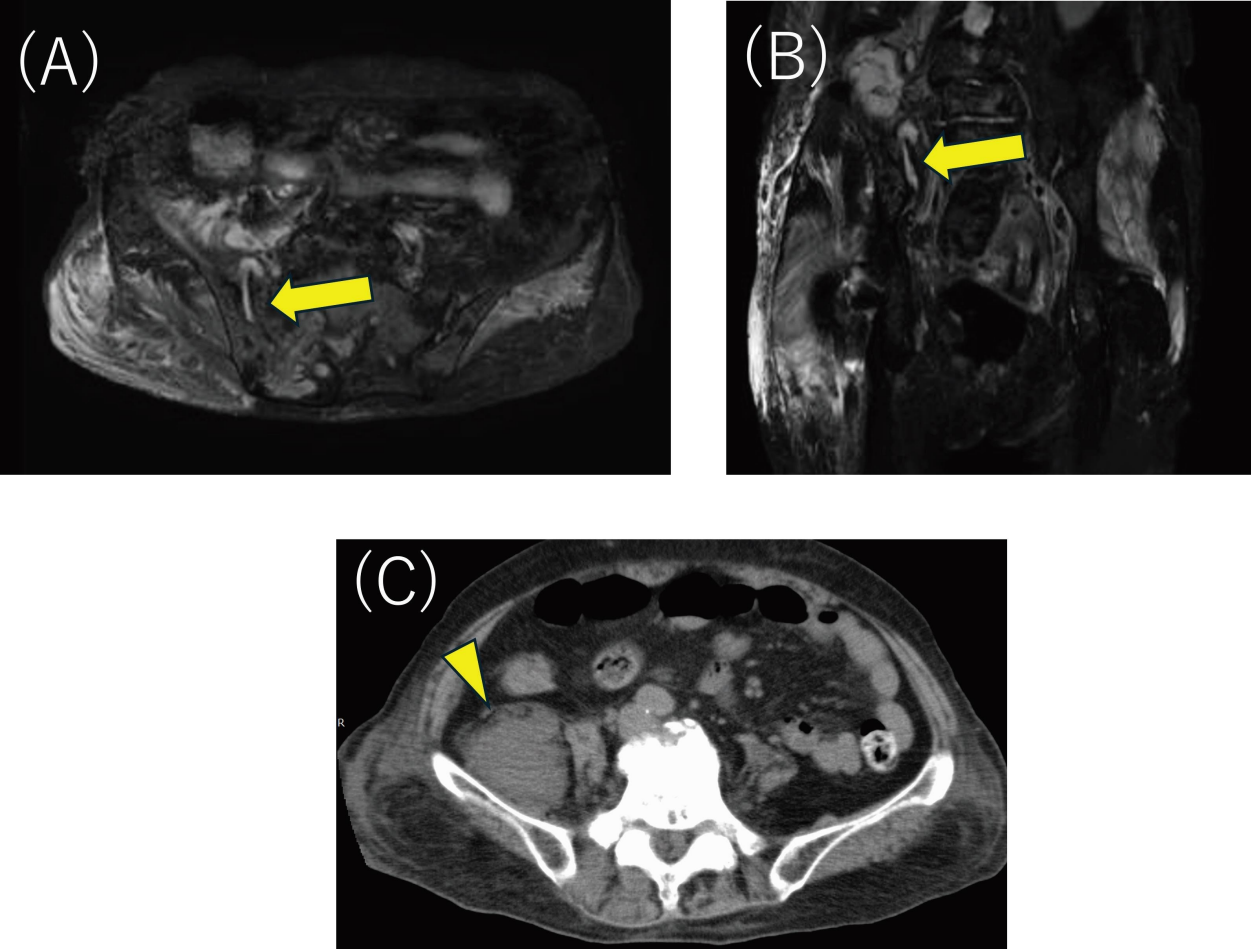
Magnetic resonance imaging (MRI) findings of the sacroiliac (SI) joint. (A) Axial and (B) coronal short‐tau inversion recovery (STIR) MRI at presentation. The right SI joint is indicated by yellow arrows, and a contiguous abscess is seen extending anteriorly into the retroperitoneal space and posteriorly into the gluteal region. (C) Axial computed tomography (CT) demonstrates a massive retroperitoneal abscess (yellow arrowhead), which initially drew attention to a possible intra‐abdominal/retroperitoneal source rather than the SI joint.

Because the massive retroperitoneal abscess was conspicuous and urinalysis showed bacteria and leukocytes, an infection of intestinal or urinary tract origin was suspected, leading to delayed recognition of PSI. Blood cultures (two sets, each containing one aerobic and one anaerobic bottle), urine cultures, and ultrasound‐guided aspiration of the gluteal abscess were performed and submitted for culture, and antibiotic treatment was initiated. After *Staphylococcus aureus* was detected in blood cultures, blood and urine cultures were confirmed to have yielded methicillin‐susceptible *S. aureus* (MSSA), and all four blood‐culture bottles became positive within 24 h, whereas the initial ultrasound‐guided gluteal aspiration was culture‐negative. Follow‐up blood cultures on Days 1, 3, and 7 remained positive, and the first documented negative cultures were obtained on Day 22. The MSSA isolates were susceptible to oxacillin and cefazolin and resistant to penicillin, ampicillin, erythromycin, clindamycin, and levofloxacin. Transthoracic echocardiography on Day 3 showed an approximately 6‐mm vegetation on the aortic valve. At 1 month, no valvular destruction was evident, and the vegetation was no longer visible. No preceding infection, skin wound, or other portal of entry was identified. Despite antibiotic treatment, her condition deteriorated with progressive renal dysfunction and altered consciousness. The initial negative gluteal aspiration, early partial improvement in pain and CRP with antimicrobial therapy, and concern that the multiple collections might reflect another primary source reduced the perceived urgency for drainage. When altered consciousness and oliguria developed around Day 19, source control became urgent. In light of this deterioration, the initial MRI was re‐evaluated, leading to the discovery of PSI and the identification of the SI joint as the primary source. Surgical drainages were sequentially performed: retroperitoneal abscess drainage via an anterolateral approach to the lumbar spine on Day 20, gluteal abscess drainage via a posterior approach to the hip joint on Day 26, and posterior thigh abscess drainage by caudally extending the Day 26 incision on Day 41. The Day 20 retroperitoneal and Day 26 posterior gluteal/hip‐region drainage specimens yielded MSSA with susceptibility profiles concordant with the Day 0 blood isolate; the Day 41 thigh specimen was negative after prolonged antimicrobial exposure. Following these drainage procedures, her condition gradually improved. Antimicrobial therapy was as follows: intravenous piperacillin/tazobactam 4.5 g every 8 h from Day 0; intravenous sulbactam/ampicillin 3 g every 8 h, ceftriaxone 2 g every 24 h, and vancomycin initially 1 g every 12 h with subsequent trough‐guided adjustment from Day 3; intravenous cefazolin 2 g every 8 h from Day 6 after MSSA was identified; intravenous sulbactam/ampicillin 3 g every 6 h plus trough‐guided vancomycin from Day 14 because CRP increased again; and intravenous cefazolin 2 g every 8 h from Day 22. Intravenous antibiotics were switched on Day 67 to oral minocycline 200 mg/day plus four combination tablets/day of trimethoprim–sulfamethoxazole, and as there was no exacerbation, she was discharged home on Day 89. Trimethoprim–sulfamethoxazole was stopped on Day 89, and minocycline was continued until Day 246. CRP had fallen to approximately 1 mg/dL by 2 months but then fluctuated between approximately 0.3 and 2 mg/dL. In view of the extensive abscesses and osseous involvement, therapy was continued until CRP normalized. MRI at 6 months showed disappearance of the abscesses and SI‐joint STIR hyperintensity. Right SI‐region pain persisted for approximately 15 months; short‐distance cane walking became possible at 5 months and stable at 7 months. Antibiotics were discontinued on Day 246 (approximately 8 months after admission), when CRP had normalized. She has had no recurrence at 2.5 years postsurgery.

### 2.2. Case 2

A 70‐year‐old woman with a history of hypertension, otitis media with effusion, and partial foot amputation at the transmetatarsal or more distal level due to a childhood burn presented with a 3‐week history of right low back and gluteal pain. She had not previously been diagnosed with any lumbar spinal disease. After developing fever and low back pain, she visited a local clinic, where she was presumed to have a common lumbar spine disorder and was treated with oral medications and analgesic injections. The oral medications and injections were analgesics; no antimicrobial agent had been administered. However, her symptoms did not improve and gradually worsened. On arrival at our hospital, she presented with fever, hypotension, and tachycardia, and was in a state of septic shock with impaired consciousness. Her vital signs were body temperature 38.0°C, blood pressure 83/64 mmHg, heart rate 126 bpm, and SpO2 93% on room air. Laboratory findings were as follows: TP 8.1 g/dL, Alb 1.6 g/dL, AST 33 U/L, ALT 21 U/L, CK 57 U/L, BUN 19.2 mg/dL, Cre 0.96 mg/dL, Na 133 mEq/L, K 5.3 mEq/L, Cl 96 mEq/L, CRP 33.98 mg/dL, glucose 201 mg/dL, HbA1c 5.8%, WBC 36,400/*μ*L, RBC 369 × 10^4^/*μ*L, Hb 11.1 g/dL, platelets 61.7 × 10^4^/*μ*L, PT‐INR 1.1, APTT 31.3 s, and D‐dimer 4.5 *μ*g/mL. Urinalysis showed RBC 5–9/HPF, WBC 10–19/HPF, and bacteria 2+. Similar to Case 1, MRI showed edema signal changes in the surrounding muscles and in the adjacent sacrum and ilium around the SI joint, along with abscess formation extending from the right retroperitoneal space to the anterior ilium and the gluteal region (Figure 2).

**Figure 2:**
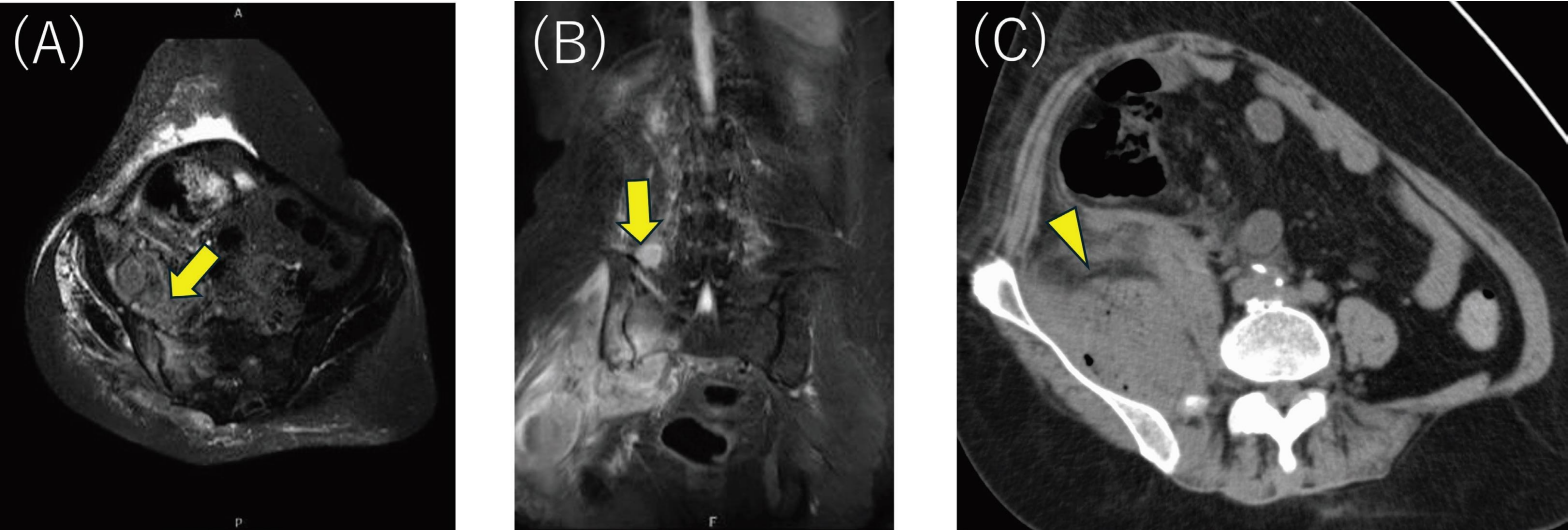
Magnetic resonance imaging (MRI) findings of the sacroiliac (SI) joint. (A) Axial and (B) coronal short‐tau inversion recovery (STIR) MRI at presentation. Yellow arrows indicate the right SI joint, with a contiguous abscess tracking from the SI joint to the retroperitoneal space anteriorly and to the gluteal region posteriorly. (C) Axial computed tomography (CT) shows a massive retroperitoneal abscess (yellow arrowhead).

Based on the experience from Case 1, PSI was immediately suspected. Before antimicrobial therapy on Day 0, two blood‐culture sets (four bottles in total) were collected, and the gluteal abscess was aspirated under ultrasound guidance and submitted for culture. All four blood‐culture bottles became positive within 24 h. Emergency irrigation and drainage of both retroperitoneal and gluteal abscesses were performed on Day 0. Blood cultures, the Day 0 gluteal aspirate, and the intraoperative anterior retroperitoneal specimen all yielded *Streptococcus agalactiae* with concordant susceptibility profiles. The isolate was susceptible to penicillin, ampicillin, ceftriaxone, clindamycin, and vancomycin. A residual thigh abscess was drained during a second operation on Day 22. For the retroperitoneal drainage, the retroperitoneal space was entered using an anterolateral approach to the lumbar spine, similar to Case 1. She required vasopressor support initially but stabilized rapidly. Follow‐up blood cultures were not obtained because prompt source control had been achieved, and the clinical and inflammatory responses indicated infection control. Therefore, microbiological clearance of bacteremia was not confirmed. Antimicrobial therapy consisted of intravenous piperacillin/tazobactam 4.5 g every 8 h plus clindamycin 600 mg every 8 h from Day 0, with clindamycin continued throughout intravenous treatment; piperacillin/tazobactam was replaced by intravenous sulbactam/ampicillin 3 g every 8 h on Day 6, and sulbactam/ampicillin was replaced by intravenous ampicillin 1 g every 12 h on Day 17. On Day 35, treatment was changed to oral clindamycin 450 mg/day plus amoxicillin 1500 mg/day, and with no recurrence of inflammation, she was discharged home on Day 52. Oral antibiotics were discontinued on Day 102 (approximately 3.5 months after admission), after CRP normalization. At the 1.5‐year follow‐up, she has had no recurrence and ambulates independently without pain. As shown in Table 1, Case 2 had a more favorable clinical course and imaging findings than Case 1 (Figure 3, Table 1). The chronological clinical courses of both patients are summarized in Figure 4.

**Table 1:** Comparison of clinical course and outcomes between Case 1 and Case 2.

Feature	Case 1	Case 2
Age and sex	83‐year‐old woman	70‐year‐old woman
Causative organism	MSSA	*Streptococcus agalactiae*
Initial CRP	31.07 mg/dL	33.98 mg/dL
Sepsis/septic shock	Bacteremia at presentation; subsequent organ dysfunction	Septic shock at presentation
Hospital stay	89 days	52 days
Number of surgeries	3	2
Procedure days	Days 20, 26, and 41	Days 0 and 22
Antibiotic duration	246 days (approximately 8 months)	102 days (approximately 3.5 months)
Ambulatory status at 6 months after admission	Walking with a cane	Independent walking
Residual symptoms (6 months)	Right gluteal pain	None
CT findings (6 months)	No osseous repair was observed	Osteosclerosis was confirmed

*Note:* Comparison of surgical burden, hospital stay, antibiotic duration, functional status at 6 months, residual symptoms, and follow‐up CT findings. All time points are defined relative to the day of presentation.

Abbreviation: CT, computed tomography.

**Figure 3:**
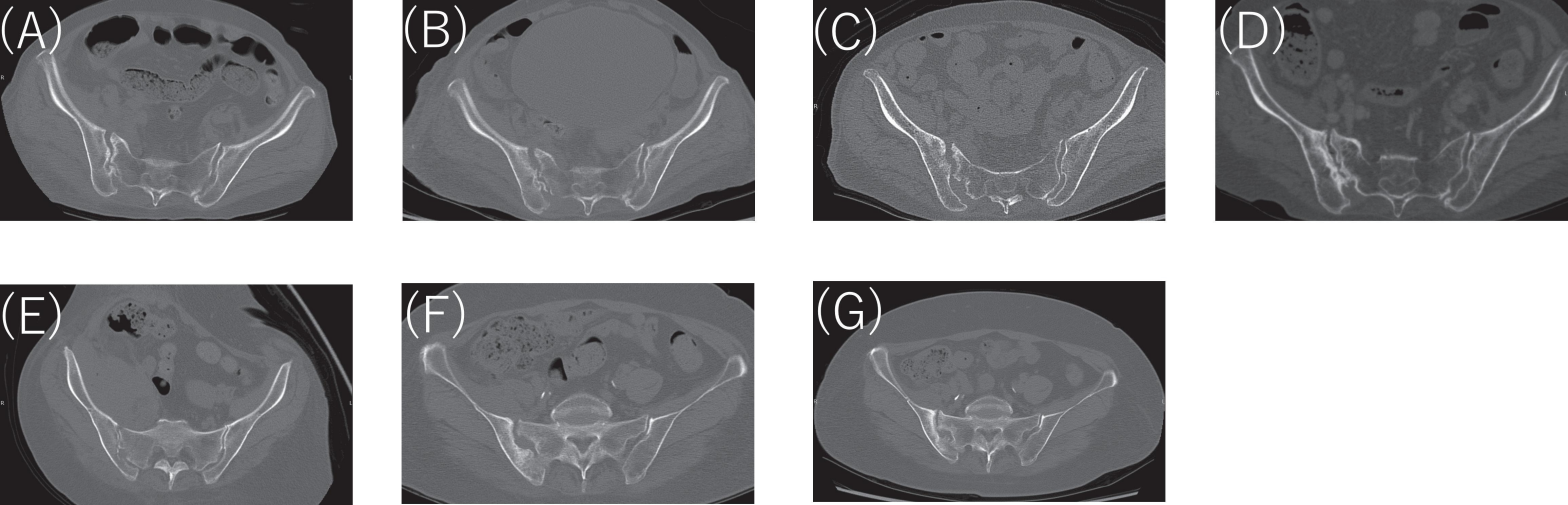
Serial bone‐window CT images showing different osseous healing patterns of the sacroiliac (SI) joint in the two cases. Serial axial CT images (bone window) of the right SI joint. (A–D) Case 1: (A) at presentation, (B) 3 months, (C) 6 months, and (D) 17 months. (E–G) Case 2: (E) at presentation, (F) 3 months, and (G) 6 months. Although osseous changes are subtle at presentation (A,E), osteolytic changes become evident at 3 months (B,F). In Case 1, osteolysis persists at 6 months (C), and osteosclerosis is only apparent at 17 months (D), whereas Case 2 shows osteosclerosis by 6 months (G), illustrating faster radiologic healing.

**Figure 4:**
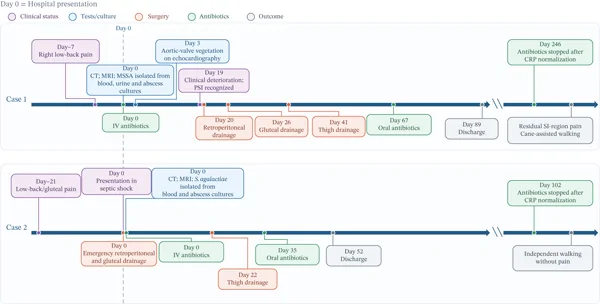
Clinical timelines of Cases 1 and 2. Day 0 represents the day of hospital presentation. Cultures identified MSSA in Case 1 and *Streptococcus agalactiae* in Case 2. Case 1 underwent drainage of the retroperitoneal, gluteal, and thigh abscesses on Days 20, 26, and 41, respectively, after clinical deterioration and recognition of PSI on Day 19. Case 2 underwent emergency drainage of the retroperitoneal and gluteal abscesses on Day 0, followed by drainage of the thigh abscess on Day 22. Purple, blue, orange, green, and gray boxes indicate clinical status, tests and cultures, surgery, antimicrobial therapy, and outcomes, respectively. Breaks in the time axes separate the early clinical course from subsequent treatment and outcome events. Abbreviations: CRP, C‐reactive protein; CT, computed tomography; IV, intravenous; MRI, magnetic resonance imaging; MSSA, methicillin‐susceptible *Staphylococcus aureus*; PSI, pyogenic sacroiliitis; SI, sacroiliac.

## 3. Discussion

PSI typically affects younger adults but can occur in older patients, often presenting atypically without high fever [5], as seen in Case 1. PSI is generally considered to begin predominantly through hematogenous seeding of the SI joint, although direct inoculation or contiguous spread from an adjacent infection can occur [2–5]. Once the SI joint is infected, subsequent extension into the retroperitoneum anteriorly and the gluteal muscles posteriorly is primarily by contiguous local spread [6]. *S. aureus* is the most frequently reported adult PSI pathogen, and *Streptococci*, including *S. agalactiae*, are also recognized causes [2–5, 7]. Thus, neither MSSA in Case 1 nor *S. agalactiae* in Case 2 was an exceptional pathogen. In Case 1, the SI‐joint–centered infection with sustained MSSA bacteremia was considered more likely to have caused secondary infective endocarditis, although hematogenous seeding of the SI joint from initially occult bacteremia or endocarditis cannot be excluded. Diagnostic recognition may be further delayed when massive abscesses extend into the retroperitoneum and divert attention from the SI joint. The average diagnostic delay is reported to be 3–5 weeks [2]. Diagnosis can be delayed even without a large abscess because low back and buttock pain are nonspecific and may mimic degenerative lumbar disease, sciatica, pyogenic spondylitis, hip disease, or abdominal disorders. The SI joint is deeply located and difficult to localize on physical examination; fever and leukocytosis may be absent; and the lesion may be difficult to identify on imaging [2–7]. Fever or elevated inflammatory markers accompanying new unilateral low back or buttock pain should therefore prompt specific review of the SI joint as well as the lumbar spine. A key anatomical feature of PSI is the potential for multidirectional spread. Inflammation can track anteriorly to the retroperitoneum, posteriorly to the gluteal muscles, and craniocaudally along the iliopsoas muscle [8, 9]. Both our cases exhibited this characteristic pattern. In both patients, MRI demonstrated anatomical continuity from the SI joint to the anterior retroperitoneal and posterior gluteal compartments. Case 1 yielded concordant MSSA from blood, retroperitoneal drainage, and posterior gluteal/hip‐region drainage; Case 2 yielded concordant *S. agalactiae* from blood, the gluteal aspirate, and the retroperitoneal operative specimen. These findings strongly support contiguous abscess extension from PSI. In Case 1, failure to recognize the SI joint as the origin of the retroperitoneal abscess led to delay. In Case 2, this specific pattern triggered immediate suspicion of PSI despite the patient′s critical state. PSI‐specific predictors of severe or fatal outcomes have not been established, likely because published series are small and heterogeneous. In native‐joint septic arthritis generally, advanced age, comorbidity, bacteremia or sepsis, and organ dysfunction are associated with poor outcomes [10]. These factors may also contribute to severity in PSI, but this remains unconfirmed and requires further study.

Although antibiotics alone can treat early PSI, surgical drainage is often considered for large abscesses [1, 5]. Antimicrobial management should include obtaining cultures before treatment whenever feasible, narrowing therapy according to the organism and susceptibility profile, draining large collections, and individualizing the intravenous‐to‐oral transition and total duration according to clinical response, CRP, abscess resolution, and osseous involvement. Delayed intervention can be fatal [2, 5]. The first drainage was performed on Day 20 in Case 1 and on Day 0 in Case 2. In Case 1, recognition of the SI joint as the primary source and surgical drainage were delayed, and clinical deterioration with renal dysfunction, altered consciousness, and oliguria occurred before source control. By contrast, in Case 2, immediate drainage of both the anterior retroperitoneal and posterior gluteal collections was followed by rapid stabilization from septic shock. This temporal contrast supports the clinical importance of prompt source control in severe PSI with extensive abscess formation, although differences in baseline characteristics and disease severity should be considered when comparing the overall clinical courses.

## 4. Limitations

This case report has several limitations. First, the portal of entry was not definitively identified, and the direction and sequence of infection, particularly the relationship among PSI, bacteremia, and infective endocarditis in Case 1, could not be established with certainty. Second, SI‐joint fluid was not directly cultured in either case. Contiguous spread from the SI joint was inferred from radiological continuity and the recovery of concordant organisms from blood and adjacent abscesses, but these findings do not provide definitive proof of the direction of spread. Third, follow‐up blood cultures were not obtained in Case 2; therefore, microbiological clearance of bacteremia was not confirmed, although clinical and inflammatory responses indicated infection control. Finally, because this report includes only two clinically heterogeneous patients, it cannot establish a causal relationship between the timing of drainage and the differences in clinical outcomes. The patients differed in age, comorbidities, causative organisms, clinical severity, extent of infection, and other aspects of treatment.

## 5. Conclusion

PSI should be considered in older patients presenting with low back pain and retroperitoneal abscesses. The presence of concurrent retroperitoneal and gluteal abscesses is a strong diagnostic clue. Timely consideration of surgical source control, together with appropriate antimicrobial therapy, may be important in severe cases with extensive abscess formation.

## Funding

No funding was received for this manuscript.

## Ethics Statement

Under the policy of our institution, formal ethics committee approval is not required for case reports or small case series describing routine clinical care without any research‐specific intervention, additional examination, or experimental procedure. Accordingly, this retrospective two‐patient case report was not submitted for formal ethics committee review. All clinical information was de‐identified. Written informed consent was obtained from the patients for publication of this case report and any accompanying images.

## Conflicts of Interest

The authors declare no conflicts of interest.

## Supporting information


**Supporting Information** Additional supporting information can be found online in the Supporting Information section. File S1: CARE checklist.

## Data Availability

The data used in this study will not be made publicly available to protect patient privacy. Upon reasonable request, they may be provided by the corresponding author to the extent that they can be anonymized.
