# Supplementary material for: Pyogenic Sacroiliitis in Older Adults Presenting With Massive Retroperitoneal Abscess: A Case Report
Source: Case Rep Orthop. 2026 Oct 17;2026:5538167. doi: 10.1155/cro/5538167 (PMC13676302; doi:10.1155/cro/5538167)
Supplement: Supplementary file 1 [file CRO-2026-5538167-s001.pdf]

CARE case report guidelines

CARE Checklist of information to include when writing a case report

(cc) BY-NC-ND

Topic	Item	Checklist item description	Reported on Line
Title	1	The diagnosis or intervention of primary focus followed by the words "case report"	1,2
Key Words	2	2 to 5 key words that identify diagnoses or interventions in this case report, including "case report"	4,5
Abstract (no references)	3a	Introduction: What is unique about this case and what does it add to the scientific literature?	31-33
	3b	Main symptoms and/or important clinical findings	33-36, 39, 40
	3c	The main diagnoses, therapeutic interventions, and outcomes	36-39, 41-43
	3d	Conclusion—What is the main "take-away" lesson(s) from this case?	44-47
Introduction	4	One or two paragraphs summarizing why this case is unique (**may include references**)	57-60
Patient Information	5a	De-identified patient specific information.	63-65, 102
	5b	Primary concerns and symptoms of the patient.	65, 104
	5c	Medical, family, and psycho-social history including relevant genetic information	63-64, 102, 103
	5d	Relevant past interventions with outcomes	
Clinical Findings	6	Describe significant physical examination (PE) and important clinical findings.	67,68, 108, 109
Timeline	7	Historical and current information from this episode of care organized as a timeline	88-100, 104-108
Diagnostic Assessment	8a	Diagnostic testing (such as PE, laboratory testing, imaging, surveys).	65-77, 112-116
	8b	Diagnostic challenges (such as access to testing, financial, or cultural)	77-81, 117-120
	8c	Diagnosis (including other diagnoses considered)	82-88, 121
	8d	Prognosis (such as staging in oncology) where applicable	
Therapeutic Intervention	9a	Types of therapeutic intervention (such as pharmacologic, surgical, preventive, self-care)	92-93, 123-124
	9b	Administration of therapeutic intervention (such as dosage, strength, duration)	93-96, 124-126
	9c	Changes in therapeutic intervention (with rationale)	
Follow-up and Outcomes	10a	Clinician and patient-assessed outcomes (if available)	96-100, 127-128
	10b	Important follow-up diagnostic and other test results	99,100, 128-132
	10c	Intervention adherence and tolerability (How was this assessed?)	
	10d	Adverse and unanticipated events	
Discussion	11a	A scientific discussion of the strengths AND limitations associated with this case report	136-146
	11b	Discussion of the relevant medical literature **with references**.	147-150
	11c	The scientific rationale for any conclusions (including assessment of possible causes)	144-146
	11d	The primary "take-away" lessons of this case report (without references) in a one paragraph conclusion	151-153
Patient Perspective	12	The patient should share their perspective in one to two paragraphs on the treatment(s) they received	
Informed Consent	13	Did the patient give informed consent? Please provide if requested	Yes ☑ No ☐
